# Effects of Polyhydroxybutyrate-co-hydroxyvalerate Microparticle Loading on Rheology, Microstructure, and Processability of Hydrogel-Based Inks for Bioprinted and Moulded Scaffolds

**DOI:** 10.3390/gels11030200

**Published:** 2025-03-14

**Authors:** Mercedes Pérez-Recalde, Evelina Pacheco, Beatriz Aráoz, Élida B. Hermida

**Affiliations:** 1 Instituto de Tecnologías Emergentes y Ciencias Aplicadas (ITECA), National Scientific and Technical Research Council (CONICET), National University of General San Martin (UNSAM), San Martin 1650, Buenos Aires, Argentina; 2 Escuela de Ciencia y Tecnología, National University of General San Martin (UNSAM), San Martin 1650, Buenos Aires, Argentina

**Keywords:** bioprinting, hydrogel, microspheres, polyhydroxyalkanoates, alginate–gelatine, tissue engineering

## Abstract

Resorbable microparticles can be added to hydrogel-based biocompatible scaffolds to improve their mechanical characteristics and allow localised drug delivery, which will aid in tissue repair and regeneration. It is well-known that bioprinting is important for producing scaffolds personalised to patients by loading them with their own cells and printing them with specified shapes and dimensions. The question is how the addition of such particles affects the rheological responsiveness of the hydrogels (which is critical during the printing process) as well as mechanical parameters like the elastic modulus. This study tries to answer this question using a specific system: an alginate-gelatine hydrogel containing polyhydroxybutyrate-co-hydroxyvalerate (PHBV) microparticles. Scaffolds were made by bioprinting and moulding incorporating PHBV microspheres (7–12 μm in diameter) into alginate–gelatine inks (4.5 to 9.0% *w*/*v*). The microparticles (MP) were predominantly located within the polymeric matrix at concentrations up to 10 mg MP/mL ink. Higher particle concentrations disrupted their spatial distribution. Inks pre-crosslinked with 15 mM calcium and containingMPat concentrations ranging from 0 to 10 mg/mL demonstrated rheological characteristics appropriate for bioprinting, such as solid-like behaviour (G′ = 1060–1300 Pa, G″ = 720–930 Pa), yield stresses of 320–400 Pa, and pseudoplastic behaviour (static viscosities of 4000–5600 Pa·s and ~100 Pa·s at bioprinting shear rates). Furthermore, these inks allow high printing quality, assessed through scaffold dimensions, filament widths, and printability (Pr > 0.94). The modulus of elasticity in compression (E) of the scaffolds varied according to the content of MP and the manufacturing technique, with values resembling those of soft tissues (200–600 kPa) and exhibiting a maximum reinforcement effect with 3 mg MP/mL ink (bioprinted E = 273 ± 28 kPa; moulded E = 541 ± 66 kPa). Over the course of six days, the sample’s mass and shape remained stable during degradation in simulated body fluid (SBF). Thus, the alginate–gelatine hydrogel loaded with PHBV microspheres inks shows promise for targeted drug delivery in soft tissue bioengineering applications.

## 1. Introduction

Hydrogels are key materials in tissue engineering and regenerative medicine due to their unique properties, which closely mimic the natural extracellular matrix (ECM). Their mechanical characteristics can be adjusted to create scaffolds that match the elastic behaviour of a wide range of soft tissues, while also influencing stem cell fate [1]. Additionally, hydrogels exhibit rheological properties that make them ideal for scaffold fabrication via moulding or bioprinting. Bioprinting, in particular, allows for the production of complex scaffold designs that enhance cell adhesion, proliferation, and differentiation [2,3]. One of the limitations of hydrogels, however, is the lack of bioactive components that actively participate in tissue repair and disease treatment. This issue has been addressed by incorporating therapeutic molecules with angiogenic, anti-inflammatory, and antimicrobial properties [4]. Polymeric microparticles (MP) loaded with bioactive agents offer several advantages, such as protection from degradation, resolution of solubility issues, and the creation of localised, sequential drug delivery systems [5], which are valuable in various medical applications of hydrogels [6,7,8,9].

The cytocompatible blend alginate–gelatine is one of the most studied hydrogels for bioprinting [10,11] since it can be easily printed due to its pseudoelastic nature, with proper printing fidelity. When the bioprinter’s ink is at 37 °C (suitable for cell viability) and the bed is at room temperature, the gelatine sol–gel transition improves printability. Cross-linking the alginate chains with a Ca^2+^ ion solution enhances the effect even further [12]. Furthermore, this biocompatible combination of protein and polysaccharide closely mimics many of the ECM and enhances cell proliferation and viability [13,14].

Polyhydroxybutyrate-co-hydroxyvalerate (PHBV) was chosen to produce microparticles due to its biocompatibility, mechanical qualities, and degradation time. PHBV, a biopolymer from the polyhydroxyalkanoates (PHA) family, is a biobased and biodegradable thermoplastic produced by some bacteria or archaea under nutrient-limited conditions in the presence of excess carbon [15,16]. Although other resorbable polymers such as polylactide (PLA), polyglycolide (PGA), and polycaprolactone (PCL) are used for biomedical applications, PHAs are preferred since it is possible to produce them via enzymatic synthesis employing microorganisms and their biodegradation rate can be controlled by the proportion of monomers (HV monomers in the case of PHBV). Furthermore, PHAs have wider applicability and the mechanical strength of PHAs can be adjusted by compounding or synthetic copolymerization to simulate the final target tissue [17,18].

PHBV is especially interesting because of its biocompatibility and slow in vivo degradation being valued because of its ability to degrade in naturally occurring metabolites without provoking acidification. For its features, several tissue engineering techniques [19], including 3D printing by filament and bioprinting [20,21], are explored with this thermoplastic.

Furthermore, the production of PHBV microparticles as microcapsules and microspheres has been thoroughly studied [22]. Due to its high cytocompatibility, they have demonstrated features as cell microcarriers showing signs of in vitro degradation after 30 days [23].

PHBV microparticles were used for the encapsulation of anti-inflammatory drugs such as flurbiprofen [24], anxiolytics such as diazepam [25], antibiotics such as rifampicin [26] and tetracycline [27], drugs to treat and prevent asthma and chemotherapy agents [28]. To facilitate drug delivery to the target site, loaded microparticles are incorporated into hydrogels. In this system, active agents encapsulated within microparticles must diffuse through two barriers given by the polymeric microparticle and the hydrogel before being released into the surrounding medium. This can reduce the initial burst release and extend the delivery duration. PHBV MP loaded simultaneously with ketoprofen, and mupirocin were incorporated in κ-carrageenan/locust bean hydrogels demonstrating a slower drug release than that of the drug loaded into the hydrogel [29]. PHBV microspheres were loaded with hydrophilic and hydrophobic compounds such as bovine serum albumin (BSA) and dexamethasone and incorporated into gellan gum [30] guaranteeing a prolonged release. An extensive evaluation of microparticles in hydrogel systems for biomedical applications was systematically reported by Carrêlo and coworkers [5].

Bioprinting enables the construction of scaffolds layer by layer; the ink that feeds the bioprinting process must meet certain requirements, with hydrogels being among the preferred materials. Rheology helps to assess hydrogels for their application in bioprinting, remarkably by characterising shear thinning behaviour, viscosity, and viscoelasticity [31,32,33].

Despite the well-known properties of alginate–gelatine hydrogels and PHBV in tissue engineering, the impact of PHBV microparticle loading on the rheology of alginate–gelatine-based inks and on the mechanical behaviour of printed scaffolds remains unexplored in the literature. Thus, the purpose of this study is to determine the influence of incorporating PHBV microparticles into the hydrogel on their rheology and the structural properties of moulded and printed scaffolds. Furthermore, by altering the concentrations of microparticles, the effort aims to determine the best range for achieving inks with excellent printability and scaffolds with proper mechanical strength. Through a detailed analysis of the relationship between microparticle content and hydrogel behaviour, this study aims to contribute to the development of advanced materials for biomedical engineering applications.

## 2. Results and Discussion

### 2.1. Microparticle Characterisation

**Figure 1:**
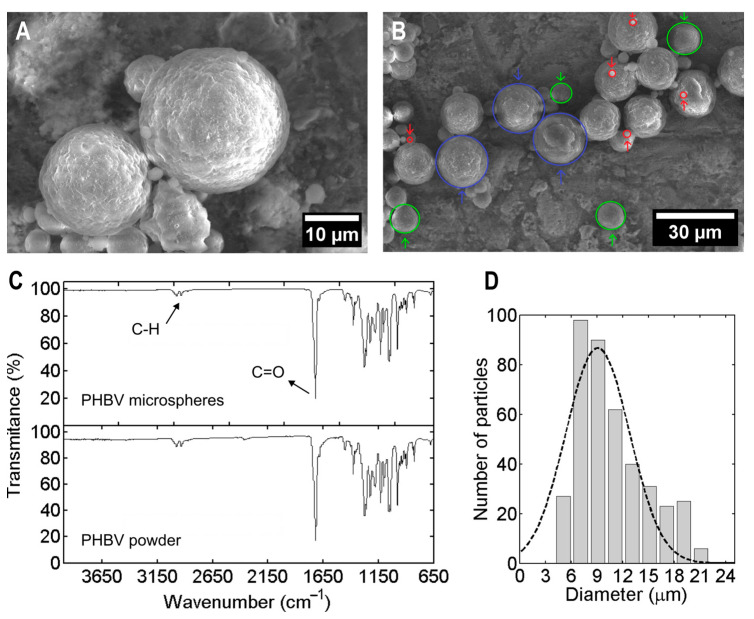
Morphological and chemical characterisation of PHBV microspheres. (**A**,**B**) Scanning electron microscopy images of lyophilised PHBV microspheres. Coloured circles depict particles with 1–3 μm diameter (red), 9–11 μm diameter (green), and 18–22 μm diameter (violet). (**C**) ATR-FTIR spectra of PHBV microspheres and PHBV powder (the arrows indicate the principal characteristic bands of PHBV). (**D**) Size distribution of microspheres (bars) and Gaussian fit (dashed line) (*n* = 415).

Figure 1A,B illustrate that the synthesised microparticles exhibited a spherical shape with a slightly rough surface and no visible pores. Figure 1C presents the ATR-FTIR spectra of PHBV powder and PHBV microspheres. The IR spectrum of PHBV powder displayed an intense band at 1720 cm^−1^, corresponding to the C=O bond stretching characteristic of the ester carbonyl group in PHAs. Bands in the spectral region around 2900 cm^−1^ were attributed to C-H stretching from methyl and ethyl groups [34,35]. The microspheres showed the same spectrum, confirming that PHBV is their sole component and that SDS was successfully removed, as indicated by the absence of a band at 3400 cm^−1^ [36]. Microspheres suspended in PBS were examined under optical (Figure S1) and electronic microscopy (Figure 1A,B); we determined a narrow size distribution with an average diameter of 9.1 ± 5.2 μm, with over 70% of the particles ranging between 7 and 12 μm. This size distribution was reproducible across six independent syntheses conducted for this study. These results agreed with the original publication from which the technique was adopted [22]. The selected particle size ensures smooth extrusion through the bioprinting nozzle, according to the guideline of being 10–100 times smaller than the nozzle diameter [37]. Moreover, PHBV microparticles of similar size have already been used for the controlled release of drugs such as flurbiprofen, highlighting its suitability for biomedical applications [24].

### 2.2. Scaffolds Produced by Moulding

**Figure 2:**
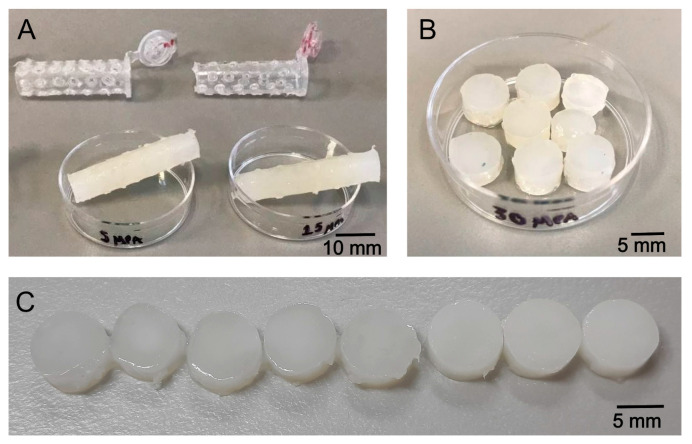
Optical images of moulded scaffolds. (**A**) Pierced Eppendorf tubes used as moulds to produce scaffolds and the crosslinked cylinders made of alginate–gelatine and PHBV microspheres; (**B**) Scaffolds produced from 5 mm thick slices cut from the cylinders, used as samples for SEM observation and compression testing. (**C**) From left to right, scaffolds were prepared with microparticle concentrations of 0, 0.5, 1.5, 3, 5, 10, 15, and 20 mg/mL ink, respectively.

Scaffolds were produced by moulding inks of alginate, gelatine, and microspheres in perforated Eppendorf tubes and cross-linking them with calcium chloride (Figure 2A). The resulting cylinders were then cut into 5 mm thick slices, as illustrated in Figure 2B,C shows scaffolds containing microspheres with concentrations ranging from 0 to 20 mg/mL ink. From left to right, the scaffolds display changes in appearance with increasing microparticle concentrations, resulting in greater light dispersion as the concentration rises.

#### 2.2.1. Microstructure Characterisation

**Figure 3:**
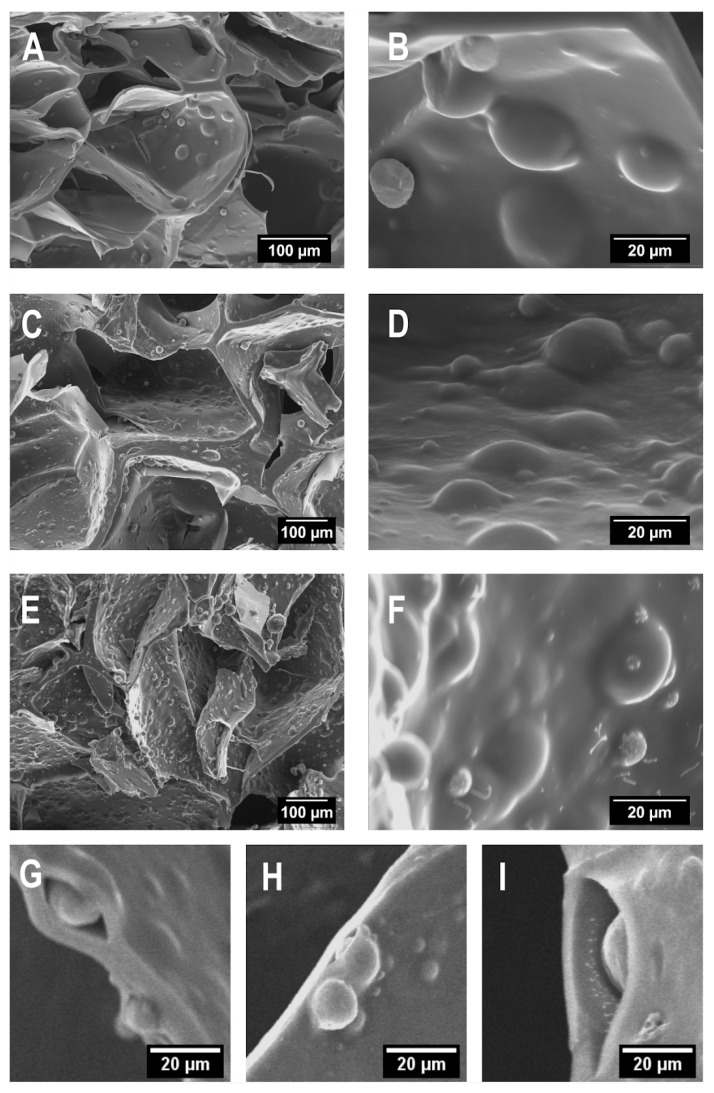
SEM images of the transversal section of scaffolds produced by moulding containing microspheres: (**A**,**B**) 3 mg/mL), (**C**,**D**) 5 mg/mL, and (**E**,**F**) 10 mg/mL ink. (**G**–**I**) Magnified images showing microspheres entrapped into the polymeric matrix of scaffolds containing 3 mg/mL ink.

To ascertain the distribution of the MP inside the scaffolds, samples were lyophilised and cut by cryofracture. Lyophilisation was used to preserve the scaffold structure since sublimation removes the water held inside the frozen crosslinked hydrogel network. Figure 3A–F corresponds to scaffolds with MP concentrations of 3, 5, and 10 mg/mL ink. The cross-section reveals pores from 300 to 500 microns with spherical protuberances, attributed to PHBV microspheres embedded into the alginate–gelatine network; in addition, scaffolds had a flat surface devoid of micron-sized pores (Figure S5). Figure 3G,I—magnification of cross-sections just in microparticle location—shows that there is no adhesion between the microparticle and the polymeric matrix, probably due to polarity differences between the hydrogel components and the PHBV. A similar behaviour was found for drug-loaded PHBV microspheres (40–80 μm) evenly distributed into gellan gum hydrogel [30].

**Figure 4:**
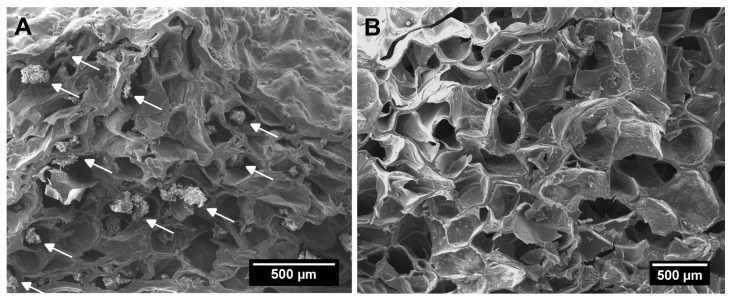
SEM images of the transversal section of scaffolds produced by moulding: (**A**) 20 mg MP/mL ink and (**B**) 5 mg MP/mL ink. White arrows indicate MP accumulation inside pores.

A different situation was observed in the case of the highest MP concentration (20 mg/mL ink) where most of the MPs were grouped out of the polymer matrix. As indicated by the arrows, Figure 4A,B shows the comparison with a scaffold containing MP in a concentration of 5 mg/mL ink, with MP embedded into the polymeric matrix as seen in Figure 3 at concentrations until 10 mg/mL.

Thus, the microparticle loading reached near maximum at a concentration of 10 mg/mL and was uniformly distributed within the polymeric matrix. Further increase in particle concentrations affected the microparticle spatial distribution.

#### 2.2.2. Modulus of Elasticity in Compression

**Figure 5:**
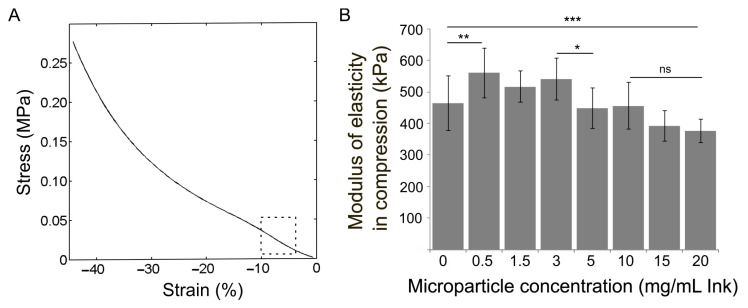
Modulus of elasticity in compression of scaffolds produced by moulding. (**A**) Representative stress vs. strain curve of a scaffold containing 3 mg/mL ink and acquired at 24 °C; the dashed square depicts the linear portion of the curve where the modulus (slope) was calculated. (**B**) Effect of the MP concentration on the scaffold modulus; bars and vertical lines represent the mean value and the standard deviation, respectively, for at least *n*  =  6 independent replicates. Horizontal lines represent one-way analysis of variance (ANOVA) with Tukey’s post hoc test for multiple comparisons. (ns: *p* > 0.05, * *p* < 0.05, ** *p* < 0.01, *** *p* < 0.001).

The mechanical behaviour of scaffolds produced by moulding was assessed using compression tests. Figure 5A shows a representative stress vs. strain curve for all moulded scaffolds: deformation at low stress due to their accommodation between the plates of the DMA clamp, followed by a stepped increase in stress at the linear viscoelastic region—where the modulus was calculated according to the standard ASTM D 1621-16 [38]—followed by a region where the hydrogel internal structure partially buckles, and finally, densification due to structure collapse [39]. Figure 5B shows the effect of MP loading on the elastic modulus. Representative curves illustrating the mechanical behaviour of each scaffold are shown in Figure S6. The modulus of scaffolds made of the neat hydrogel was 460 ± 86 kPa; it is attributed to physical crosslinking due to the electrostatic interaction between alginate chains and calcium ions, which helps to preserve the microstructure of the porous scaffold [40]. Due to MP loading during the scaffold fabrication, the modulus increased from 460 ± 86 kPa (neat hydrogel) to 560 ± 78 kPa (0.5 mg/mL ink). This reinforcement effect agreed with the microstructure observed in SEM images. The structural stiffness of the hydrogel was significantly enhanced by cross-linking with calcium ions and was further complemented by the incorporation of PHBV microspheres. This material, known for its elastic modulus close to 1.3 GPa [41,42] provides a notable improvement in the mechanical properties of the hydrogel, increasing its modulus of elasticity in compression. The reinforcement effect is preserved in scaffolds containing MP until 3 mg/mL ink (E = 541 ± 66 kPa). Further incorporation of MP revealed a deleterious effect on the mechanical properties. The scaffold modulus decreased significantly at concentrations above 5 mg/mL ink. SEM images of scaffolds containing MP at a concentration of 10 mg/mL showed a polymeric matrix fully packed with PHBV microspheres that probably affected the weak electrostatic interaction of alginate chains and calcium ions, leading to a decrease in the crosslinking degree, and the saturation of the hydrogel matrix. The interplay of both effects produced scaffolds with a modulus close to that of the neat hydrogel (E = 456 ± 74 kPa for a MP content of 10 mg/mL ink). A further increase in MP concentration was no longer supported by the hydrogel network. The scaffolds containing MP above 15 mg/mL ink presented a modulus in compression below that of the neat hydrogel. SEM images of scaffolds with 20 mg/mL showed PHBV microspheres out of the hydrogel network and suspended in the pores (Figure 4B). Through these mechanisms, the reinforcing effect of the microspheres is diminished. Moreover, even though MPs were almost completely dispersed in the pores, the modulus of scaffolds was lower than that of the neat hydrogel (E = 376 ± 37 kPa for 20 mg/mL). This observation suggests that the expulsion of microspheres from the polymeric network directs the hydrogel’s microstructure toward a new configuration with diminished electrostatic interactions. PHBV microsphere concentration showed a strong effect on the mechanical properties of the alginate–gelatine hydrogel, suggesting that by controlling the concentration of PHBV MP, the mechanical behaviour of the scaffold can be finely adjusted. This capability is particularly crucial in biomedical engineering, where precise mechanical properties can influence cellular fate [1]. This MP reinforcement was also observed for chemically cross-linked 6% gelatine scaffolds loaded with 30 mg/mL PLA microspheres of 0.5 μm in diameter [43] and for gellan gum and gelatine methacrylate scaffolds loaded with 50 mg/mL of 120 μm PLA microspheres [44].

### 2.3. Inks Containing PHBV Microspheres: Rheological Characterisation

The rheological behaviour of the inks, both in oscillatory and flow modes, was assessed to evaluate the effects of microparticle incorporation within the hydrogel and to determine their potential performance as inks for bioprinting. According to results in Section 2.2, it was decided that the microparticle concentration should be up to 10 mg/mL ink, i.e., not including MP in concentrations that negatively affect the modulus of elasticity in compression in the final scaffolds. The alginate–gelatine base ink, at the proposed concentrations, was extensively studied and deemed suitable for bioprinting [11,40]. In this sense, a pre-crosslinking with 15 mM calcium was found to be suitable for optimising its printability (Supplementary Data Figures S2 and S3). Pre-crosslinked inks+MP were named ink-0.5 to ink-10 according to their MP concentrations.

**Figure 6:**
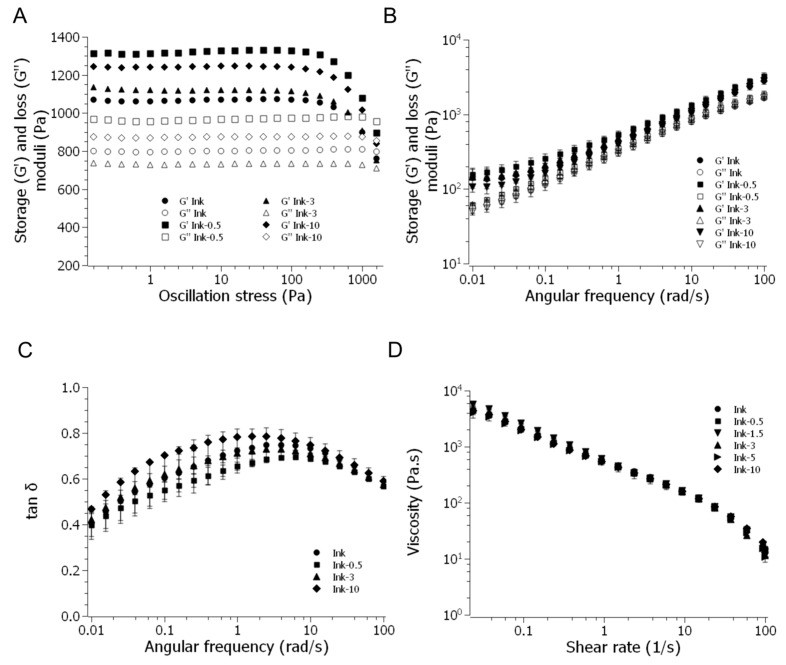
Rheological analysis of ink (circles), ink-0.5 (squares), ink-3 (triangles), and ink-10 (diamonds): (**A**) Storage and loss moduli as a function of oscillation stress; the arrows indicate the yield stress. (**B**) Storage and loss moduli vs. frequency and (**C**) Loss tangent vs. frequency (**D**) Viscosity as a function of shear rate including intermediate concentrations ink-1.5 (inverted triangles) and ink-5 (horizontal triangles).

To characterise the material’s viscoelastic behaviour the storage (G′), and loss (G″) moduli of inks with different MP content were determined from amplitude sweeps (Table 1). In all cases, a solid-like behaviour (G′ > G″) and LVR between 0.01 and 10% were observed. All of the inks showed comparable values for G′ and G″, with the exception of ink-0.5, which had the lowest microparticle content and a little greater modulus than the others. From these sweeps the yield stress was obtained, where moduli were plotted as a function of oscillation stress (see Table 1 and Figure 6A).

Yield stress is a relevant parameter to assess materials for bioprinting. It is the stress at which G′ starts dropping significantly, indicating the transition from solid-like to liquid-like behaviour. A minimum of around 100 Pa is recommended for bioprinting with shape fidelity [32]. Thus, all inks are suitable for bioprinting.

**Figure 7:**
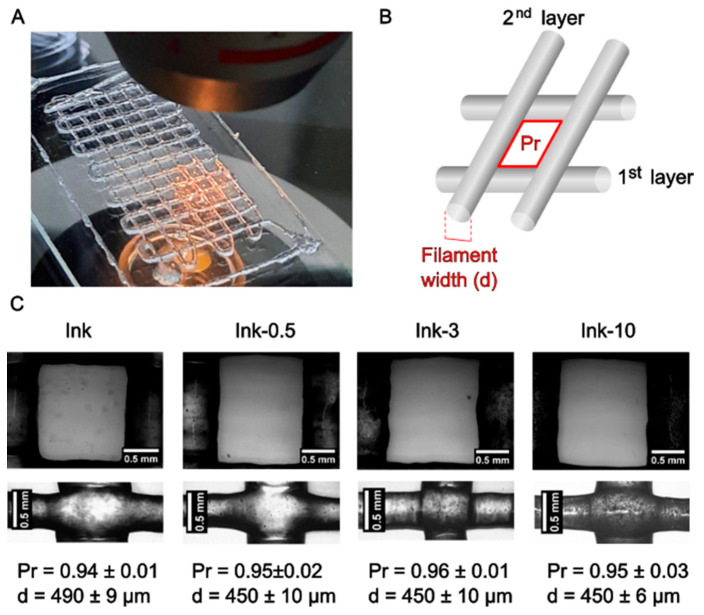
(**A**) Two layers alginate–gelatine-MP bioprinted grid. (**B**) Scheme of the printed grid. The red square depicts the area and perimeter where the Pr was calculated. (**C**) Optical images (400×) of printed squares and filaments of the second layer for different PHBV microparticle contents: from left to right ink, ink-0.5, ink-3, and ink-10. *n* = 10 to 15 replicas for Pr and *n* = 4 to 6 for filament widths.

Figure 6B shows storage and loss moduli as a function of angular velocity—frequency sweeps, that give information about hydrogel structure and dynamic response—for ink-0.5, ink-3 and ink-10 in comparison with Ink. Inks increased their modulus along the different frequencies. Similar sweeps were obtained for ink-1.5 and ink-5 (Supplementary Data Figure S4). Thus, the presence of MP does not affect the hydrogel structure or its dynamic response. Again, like in strain sweeps, only ink-0.5 has a slightly higher modulus than the rest of the inks (Figure 7, squares). Figure 6C shows the dependence of the loss tangent (tan δ) with frequency, with close values for all the inks and mainly in the range 0.35–0.75, range associated with suitable printability for alginate–gelatine systems [11].

The viscosities of all the inks subjected to varying shear rates are shown in Figure 6D. Even at the greatest concentration (ink-10), the inclusion of MP did not affect the shear-thinning behaviour. Particles did not affect the ability to be extruded in a bioprinter syringe when considering shear rates larger than 1 1/s, which is consistent with a bioprinting process. Table 2 displays viscosities at 20 1/s as an illustration of the extrusion in our work and under circumstances that are more akin to a static state. For complex viscosities, frequency sweeps revealed the same pseudoplastic behaviour (not depicted).

### 2.4. Printability

We selected three inks to assess their performance for bioprinting: ink-0.5, ink-3, and ink-10 being the lowest, intermediate, and highest MP content. Once the grid design was bioprinted, pictures were taken in an optical microscope, and from which square areas and perimeters were measured to calculate Pr and filament widths (Figure 7B). It is important to note that the second layer is from which filament widths were considered: the first layer, according to the setup in the bioprinter, has no homogeneity in height, causing wider filaments than those of the design. In fact, the small differences in filament widths among inks may be attributed above all to inherent aspects of the bioprinting technique (room temperature fluctuations, first layer height, and internal nozzle diameter variability).

Given that the inner diameter of the 21G needle employed in the bioprinter was approximately 514 µm, there was a tendency for the MP-containing inks to somewhat constrict after printing. According to the rheological evaluation, all inks had comparable viscosities at the printing shear rates, and adding MP up to 10 mg/mL ink had no effect on the printing quality. These findings corroborated rheological testing, which showed that inks with and without particles had comparable results for tan δ, yield stress, and viscosity.

### 2.5. Characterisation of Bioprinted Scaffolds

**Figure 8:**
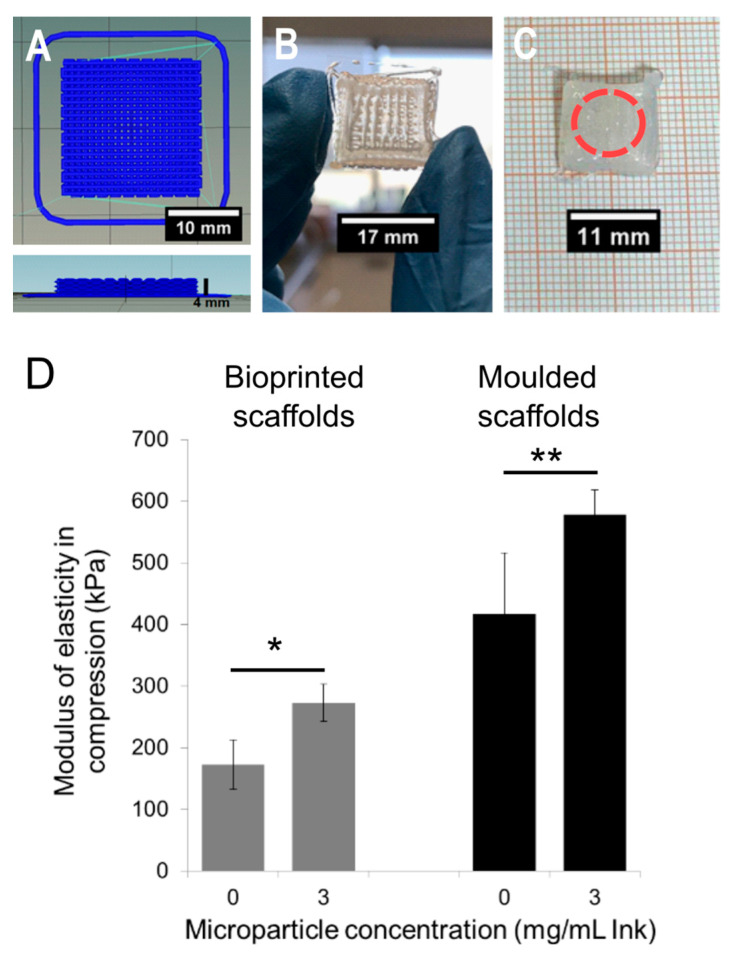
Morphology and modulus of elasticity in compression of bioprinted scaffolds. (**A**) Digital design of the scaffold with rectilinear pattern and 30% infill. (**B**) Optical image of the bioprinted structure made of ink-3. (**C**) Scaffold after being crosslinked with CaCl_2_. (**D**) Modulus in compression of printed scaffolds made with ink and ink-3 (grey bars) compared with the corresponding moulded scaffolds (black bars). Horizontal lines represent one-way analysis of variance (ANOVA) with Tukey’s post hoc test for multiple comparisons (* *p* < 0.05, ** *p* < 0.01).

Scaffolds were fabricated by a bioprinter choosing a squared design with eight layers and 30% infill (Figure 8A–C). Even though inks with all the MP concentrations demonstrated being printable, we selected the concentration 3 mg/mL ink, the highest amount with reinforcement impact on mechanical properties of scaffolds fabricated by moulding; in addition, it is a commonly used concentration in the literature for studies involving controlled release [29]. Thus, cylindrical discs for compression tests were obtained from crosslinked scaffolds made with ink-3 along with Ink, to evaluate the impact on mechanical properties through this fabrication method. Figure 8D shows that printed scaffolds exhibited an increase in the modulus of elasticity in compression due to MP content, rising from 173 kPa ± 40 kPa (Ink) to 273 kPa ± 28 kPa (ink-3). Representative strain vs. stress curves are presented in Figure S7. Moreover, the moduli were lower compared to scaffolds produced by moulding, primarily due to microstructural differences due to channels created during the bioprinting process. In this regard, the reinforcing effect of MP was more pronounced in bioprinted scaffolds, with an increase of approximately 60%, compared to about 17% in moulded samples. The bioprinting technique allows for this additional control over the mechanical properties based on the infill percentage and infill pattern [45].

**Figure 9:**
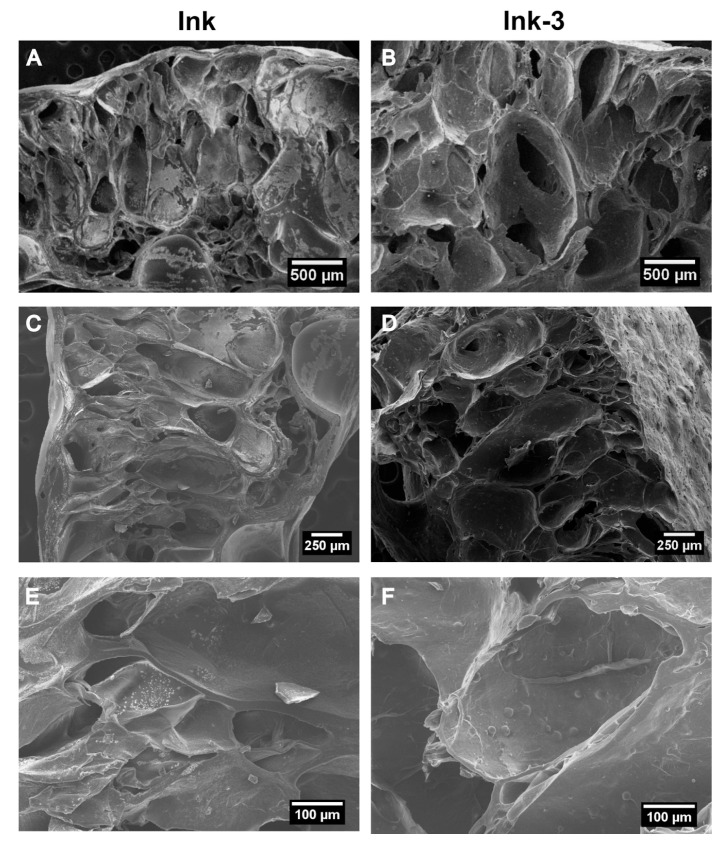
SEM images of the transversal section of bioprinted scaffolds with Ink (**A**,**C**,**E**) and with ink-3 (**B**,**D**,**F**). Channels due to printed design can be observed in addition to typical hydrogel pores.

Figure 9 illustrates two distinct pore distributions: first, the big pores associated with the bioprinting process, forming ellipsoidal channels with their major axis oriented perpendicular to the printing bed, ranging in size from 600 to 900 μm. Second, the pores typical of the hydrogel’s porous matrix, with sizes between 200 and 500 μm, are comparable to those generated by moulding. Additionally, bioprinted scaffolds exhibited MP homogeneously distributed, as in the moulded scaffolds, contributing to enhanced mechanical performance.

### 2.6. Degradation in Simulated Body Fluid (SBF)

We assessed the stability of scaffolds at 37 °C in simulated body fluid (SBF). The choice of SBF for the incubations was based on its ionic composition, which closely resembles that of biological fluids; in addition, its calcium concentration is particularly relevant for the stability derived from the crosslinking method [46]. Moreover, preliminary results using PBS as the incubation medium showed that the scaffolds lose their manipulability and structural integrity, ultimately disintegrating by day three. This phenomenon can be attributed to the leaching of calcium ions from the scaffold, which previously acted as crosslinking agents stabilising the hydrogel structure, thereby reinforcing the use of SBF as a more suitable medium for evaluating the in vitro stability of the scaffolds [40,47].

**Figure 10:**
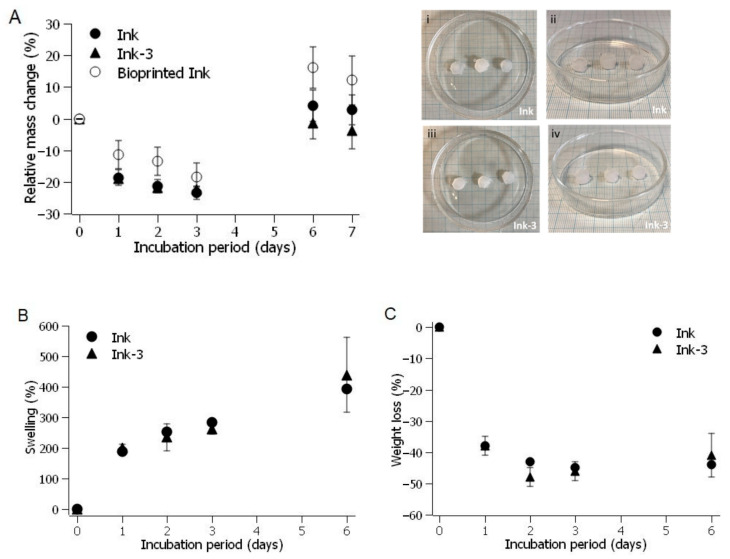
Stability of moulded and bioprinted scaffolds in SBF at 37 °C. (**A**) Relative mass change in scaffolds incubated for 7 days. Images i–iv show moulded scaffolds after 6 days of incubation, without (i,ii) and with (iii,iv) microparticles. (**B**) Effect of microparticles on swelling of moulded scaffolds incubated in SBF at 37 °C for 6 days. (**C**) Effect of microparticles on weight loss of moulded scaffolds incubated in SBF at 37 °C for 6 days.

Figure 10A illustrates the relative mass change over time of scaffolds, with a medium refresh performed on the third day of incubation. Moulded scaffolds with and without microspheres and bioprinted scaffolds exhibited a similar profile, a negative mass change until the medium renewal. The relative mass change turns positive after the buffer change (from the third to the sixth day). These changes do not affect their appearance in shape and structure, which remains comparable to the initial scaffolds (Figure 10Ai–iv).

To address potential stability issues and explore the feasibility of fabricating bioinks with cells incorporated into the polymer mixtures, we tested the same compositions using PBS as the solvent instead of water for the polymers employed in this study [48]. Both moulded and bioprinted scaffolds prepared in PBS exhibited similar trends to those produced with water as the solvent, indicating no significant impact on SBF incubations (Figure S8).

The relative mass changes observed in the hydrogels result from the interplay of two opposing phenomena occurring simultaneously: water absorption that induced swelling and material degradation [49]. Scaffolds fabricated through moulding with and without microparticles were used as a reference to evaluate the contribution of each process. Figure 10B illustrates the swelling behaviour in lyophilised scaffolds. The scaffolds swelled up to 200% of their initial weight within the first day of incubation. This water absorption causes a minimal change in the scaffold diameter (less than 6%), indicating that the absorbed water is primarily accommodated within the pores of the lyophilised scaffold thereby hydrating the polymer chains. Following medium renewal, the swelling increases again, as previously observed in Figure 10A. Microparticles did not alter their water absorption capacity. The degradation of the scaffolds (Figure 10C), measured as weight loss (%), was 38 ± 2% on the first day, denoting the onset of degradation in the tested scaffolds. By the sixth day, the weight loss had slightly decreased. The initial weight loss can be attributed to the dissolution of the gelatine [47]. This behaviour was consistent across all tested scaffolds, likely due to minimal alginate loss, which helped preserve the scaffold’s shape.

## 3. Conclusions

In this work, we examine how the concentration of polymeric microspheres affects the rheology and printing quality of hydrogels inks, as well as the mechanical properties of scaffolds produced through moulding and bioprinting. The viscosity of the inks has virtually no dependence on the MP concentration. The evaluation of these materials for bioprinting inks is of relevance to this feature, particularly in local drug release and tissue regeneration. Excellent reproducibility and manipulability have been demonstrated by scaffolds made by bioprinting and moulding. Because of the combination of alginate and gelatine, the scaffolds produced by moulding had a porous structure. Up to 10 mg/mL ink, the microspheres were evenly dispersed mostly within the hydrogel’s polymeric matrix.

Both the microparticle content and the fabrication method impact on the mechanical properties of the scaffolds under compression. Due to the microspheres’ reinforcing impact within the polymeric matrix, moulded scaffolds showed a rise in modulus in compression (15–20%) when microparticle loading reached 3 mg/mL. The polymeric matrix was disrupted by higher MP concentrations (15 or 20 mg/mL ink), which reduced the modulus.

Production techniques and microparticle compositions could help adjust mechanical qualities based on the target tissue. Specifically, microspheres perform two functions: they tune the mechanical characteristics of the scaffold and allow for regulated drug release. Scaffolds made by bioprinting or moulding display all modulus in compression (170–570 kPa) found in a variety of soft tissues, such as muscle, skin, and the lungs [50]. Because the internal pattern design and infill % in the bioprinting software allow for control over internal pore size and channels, scaffolds made by moulding have a higher modulus than those made by bioprinting. These production variables control how the scaffold behaves mechanically to orient itself toward the patient’s tissues.

Increasing the microparticle load to enhance the released dose compromises the system’s mechanical properties and potentially alters the release profile due to disruption of the hydrogel network and the heterogeneous distribution of microspheres. Notably, the concentration used in various drug delivery systems (DDSs) aligns with the maximum reinforcement threshold of our system (3 mg/mL), suggesting that balancing reinforcement and controlled release could be a critical factor in optimising the formulation. In this work, composite inks with microparticle concentrations above 5 mg/mL ink result in matrix saturation, which compromises the stiffness and microstructure of the composite material. With 3 mg/mL ink, scaffolds have a similar degradation and swelling profile in SBF than pristine alginate–gelatine.

Since PHBV microspheres have been shown in previous studies to be effective for encapsulating and delivering active molecules, the resulting scaffolds are of interest for applications in soft tissue bioengineering. These findings highlight the potential of incorporating microparticles to tailor the mechanical and manufacturing properties of polymeric scaffolds, paving the way for future advancements in sequential drug delivery systems for tissue engineering applications.

## 4. Materials and Methods

### 4.1. Materials

The polyhydroxybutyrate-co-valerate, PHBV (ENMAT Y1000 1.5% mol HV), was obtained from TianAn, China. Gelatine 250 Bloom, commercial food-grade, and calcium alginate commercial food-grade were obtained from a distributor by Van Rossum S.A., Buenos Aires, Argentina. Reagents to prepare buffers and solutions were analytical grade and dissolved in MilliQ water. All chemicals were analytical grade used without further purification.

### 4.2. Microspheres Synthesis and Characterisation

Polymeric microspheres were synthesised by simple emulsion oil/water and solvent evaporation according to Farrag [15] under the conditions 1% *w*/*v* PHBV and 1% *w*/*v* SDS. Briefly, 0.5 g of PHBV were dissolved in 50 mL of dichloromethane under magnetic stirring at 1000 RPM for 3 h; later, sonication cycles at 40 °C for 20–30 min were repeated 4 times. A solution of SDS (1 g) in water (100 mL) was placed under magnetic stirring (1000 RPM) and the PHBV solution was added. After 1 h, the emulsion was placed in a water bath at 42 °C for ca. 2 h, time required for the complete dichloromethane evaporation. Four washes with distilled water were made by centrifugation at 3500 rpm, at 20 °C for 10 min. MP were freeze-dried, and stored until its use.

Optical microscopy images of microspheres suspended in PBS (Figure S4) were analysed in ImageJ software [51] version 1.542 for particle size determination (*n* = 415). The size distribution histogram was obtained according to the Sturges rule and fitted by a Gaussian function to determine the average size and standard deviation. All subsequent synthesis were controlled by optical microscope images to confirm method reproducibility. Microsphere morphology was evaluated in a scanning electron microscope (SEM) Quanta 250, FEI, Quanta, Hillsborn, OR, USA, after coating with a thin layer of Au-Pd (40:60).

The chemical characterisation of PHBV microspheres was evaluated by FTIR-ATR. Infrared spectra were obtained on an FTIR Nicolet 8700 (Thermo Scientific, Waltham, MA, USA spectrophotometer with a ZnSe crystal, 32 scans, and a resolution of 4 cm^−1^.

### 4.3. Inks Preparation

The inks were prepared by mixing gelatine and alginate powders in Milli-Q water, followed by incubation in a water bath at 37 °C for 20 min to dissolve the polymers. To remove air bubbles, the inks were centrifuged for 5 min at 5000 rpm and then kept at 37 °C until use. The final polymer concentrations were 4.5% *w*/*v* gelatine and 9% *w*/*v* alginate. Inks containing microspheres (MP) were produced by adding MP to the polymer powders (0.5–10 mg of MP per millilitre of ink) and following the same preparation process.

For inks prepared for bioprinting, a CaCl_2_ solution was added to the MilliQ water at a final concentration of 15 mM to pre-crosslink the ink, thereby increasing its stiffness and printing quality. The CaCl_2_ concentration was selected according to the results presented in Figures S1 and S2.

### 4.4. Characterisation of Inks

#### 4.4.1. Rheology

Ink rheological behaviour was assessed using a Discovery HR-3 rheometer (TA Instruments, New Castle, DE, USA) equipped with a 40 mm diameter parallel-plate geometry and setting a 1000 µm gap. Before each measurement, samples were loaded at 37 °C and allowed to stabilise for 180 s. The linear viscoelastic range (LVR) was determined by conducting amplitude sweeps (from 0.01% strain to 100% strain) at a frequency of 10 rad/s. Frequency sweeps were then performed from 100 rad/s to 0.01 rad/s at a strain of 1%. Additionally, flow sweeps were conducted over a range of shear rates (from 0.015 1/s to 100 1/s). All measurements were conducted at 37 °C. Storage (G′) and loss (G″) moduli were recorded, with careful monitoring of the raw factor to ensure data integrity.

#### 4.4.2. Printing Quality

Printing quality was assessed using printability (Pr), filament width, and dimensions of bioprinted scaffolds. To determine Pr a grid design with 48 squares was chosen. Grids were bioprinted in a LIFE SI 3D-Res bioprinter (Córdoba, Argentina) equipped with a 21G needle (nozzle internal diameter ~514 μm). The rectilinear model was designed in FreeCad version 0.21.1 with 30 × 15 × 1 mm dimensions and converted into a .stl file. The model was processed in Slic3r version 5.0.3 defining the printing parameter infill as 10% and the .gcode generated. Finally, the .gcode file was used in the bioprinter software where the flow was set at 60–65 μL/min, the syringe cartridge temperature set at 37 °C and the bed temperature 20 °C. Optical images of printed grids were acquired in a Mikoba microscope (Microvision, Santa Fe, Argentina) and Pr was calculated as

Pr = P^2^/16A (1)

where P is the internal square perimeter (see scheme in Figure 7B) and A is the area of the square. Optical images were used to measure the filament width from the second bioprinted layer using ImageJ software version 1.542. Bioprinted scaffold dimensions were measured using a digital calliper.

Shear rate ($\dot{\gamma }$) in relation with printing conditions was calculated according to Equation (2) [52] where R is the needle radio and V and t, volume and time, respectively.

(2) $$\dot{\gamma }=1/R^{3}\times \Delta V/\Delta t$$

### 4.5. Fabrication of Scaffolds by Moulding

Scaffolds created via moulding were fabricated by crosslinking through immersion in 0.5 M CaCl_2_ solution. The container was a perforated Eppendorf tube, facilitating the diffusion of calcium ions into the ink mixture. A series of incubation periods were implemented to ensure maximum reproducibility among the fabricated scaffolds. Initially, an incubation period of 4 h at 37 °C was employed, followed by 14 h at 8 °C. After incubation, the scaffolds were carefully removed from the tubes and transversely sliced into cylinders of 5 mm in height for subsequent measurements.

### 4.6. Fabrication of Scaffolds by Bioprinting

Scaffolds were designed with 10 mm × 10 mm × 3 mm (width × length × height, 8 layers) dimensions with an infill of 30% and a rectilinear pattern. The printing conditions matched those used for the grid in the printing quality assessment, and the crosslinking conditions were identical to those applied to scaffolds fabricated by moulding.

### 4.7. Characterisation of Scaffolds Mechanical Properties and Morphology

Mechanical properties of scaffolds produced by moulding and bioprinting were evaluated by compression test using a DMA Q800 (TA Instruments, New Castle, DE, USA). Experiments were performed at 1 mm/min with a preload force of 0.1 N. The shape of the samples was cylindrical, 7 mm in diameter and 5 mm in height for moulded scaffolds and 7 mm in diameter and 3 mm in height for the bioprinted scaffolds. The engineering compressive stress was calculated by dividing the applied force by the mean cross-sectional area of each scaffold, determined just before starting the measurement. The modulus of elasticity in compression was calculated from the linear region of the stress vs. strain curves according to ASTM D 1621-16 [38].

The internal structure of scaffolds was explored by scanning electron microscopy (Philips XL30 Serie 30 and Quanta 250, FEI, Quanta, Hillsborn, OR, USA). Samples were freeze-dried, cryogenically cut in liquid nitrogen, and coated with a thin layer of Au–Pd (40:60).

### 4.8. Determination of Changes on Scaffolds Weight After Incubation in SBF

Simulated Body Fluid (SBF) was prepared according to Kokubo et al. [46]. Scaffolds were fabricated in the same manner as described in Section 4.5 and Section 4.6 (*n* = 3–4) to assess in vitro degradation. Both moulded scaffolds (dimensions: 7 mm diameter and 5 mm height) and bioprinted scaffolds (11 mm per side and 3 mm in height) were incubated in a thermostatic bath (model HH-S4, TestLab, Buenos Aires, Argentina) at 37 °C (pH = 7.40) in a volume of 3 mL SBF/sample, for periods of 0, 1, 2, 3, and 6 days. The incubation medium was refreshed on the third day. The initial weight (m_0_) and weights at the different incubation times (m_t_) were determined by removing excess solution from the samples (*n* = 4) using absorbent paper. The percentage change in mass at the relevant time points was calculated according to Equation (3).

(3) $$Relative\;mass\;change\;(\%)=\frac{{(m}_{t}-m_{0})}{m_{0}}\cdot 100\%$$

### 4.9. Weight Loss and Swelling Degree of Freeze-Dried Scaffolds After Incubation in SBF

Moulded scaffolds without and with 3 mg/mL ink of microparticles, size 7 mm diameter and 5 mm height, were freeze-dried, and incubated in SBF as described in Section 4.8. The initial weight (w_0_) was determined before incubation. Weights in the swollen state at the different incubation periods (s_t_) were determined by removing excess solution from the samples using absorbent paper. To determine the remaining mass of scaffolds after incubation the scaffolds were removed from the incubation medium, freeze-dried, and weighted (d_t_). The swelling degree and weight loss (%) at 1, 2, 3, and 6 days of incubation were calculated according to Equations (4) and (5), respectively.

(4) $$Swelling\;degree\;(\%)=\frac{{(s}_{t}-w_{0})}{w_{0}}\cdot 100\%$$

(5) $$Weight\;loss\;(\%)=\frac{{(d}_{t}-w_{0})}{w_{0}}\cdot 100\%$$

### 4.10. Statistical Analysis

Data are presented as mean ± standard deviation. One-way analysis of variance (ANOVA) with Tukey’s post hoc test for multiple comparisons (ns: *p* >  0.05, * *p*  <  0.05,** *p*  <  0.01,*** *p* <  0.001).

## Figures and Tables

**Table 1:** Storage and loss moduli and yield stress at 37 °C and 1 Hz. Two replicas were considered for each data.

	Ink	Ink-0.5	Ink-1.5	Ink-3	Ink-5	Ink-10
G′ (Pa)	1092 ± 31	1326 ± 1	1184 ± 55	1059 ± 88	1097 ± 118	1164 ± 116
G″ (Pa)	807 ± 8	923 ± 65	847 ± 48	726 ± 10	766 ± 20	800 ± 107
Yield Stress (Pa)	336 ± 10	398 ± 9	367 ± 9	315 ± 12	327 ± 13	347 ± 27

**Table 2:** Viscosities at rest and at bioprinting conditions, for the different inks containing PHBV microspheres.

	Ink	Ink-0.5	Ink-1.5	Ink-3	Ink-5	Ink-10
Viscosity (Pa.s) at 0.02 1/s	4521 ± 813	4488 ± 583	5635 ± 311	4813 ± 825	3958 ± 356	4425 ± 328
Viscosity (Pa.s) at 20 1/s	100 ± 10	97 ± 9	101 ± 3	98 ± 6	99 ± 1	101 ± 8

## Data Availability

The original contributions presented in this study are included in the article and Supplementary Materials. Further inquiries can be directed to the corresponding authors.

## Supplementary Materials

The following supporting information can be downloaded at: https://www.mdpi.com/article/10.3390/gels11030200/s1, Figure S1: Examples of optical images used to determine diameter particle size; Figure S2: Rheological comparison between Alg-Gel 9–4.5% and Alg-Gel 9–4.5% prescrosslinked with calcium 15 mM; Figure S3: Evaluation of Pr Ink Alg/Gel with different level of calcium precrosslinking (2.5–40 mM); Figure S4: Frequency sweeps for ink-1.5 and ink-5; Figure S5: SEM image of cross-section of liofilized moulded scaffold (produced with ink-3) after cryogenic fracture; Figure S6: Representative stress vs. strain curve of scaffolds produced by moulding of inks containing different concentrations of MP: 0 mg MP/mL (ink), 0.5 mg MP/mL (ink-0.5), 1.5 mg MP/mL (ink-1.5), 3 mg MP/mL (ink-3), 5 mg MP/mL (ink-5), 10 mg MP/mL (ink-10), 15 mg MP/mL (ink-15), and 20 mg MP/mL (ink-20); Figure S7: Representative stress vs. strain curve of scaffolds produced by bioprinting inks containing different MP concentrations: 0 mg MP/mL (ink) and 3 mg MP/mL (ink-3); Figure S8: Stability in SBF at 37 °C of moulded and bioprinted scaffolds using PBS or water as solvent evaluated as the relative mass change for 7 days.
