# Supplementary material for: Effects of Polyhydroxybutyrate-co-hydroxyvalerate Microparticle Loading on Rheology, Microstructure, and Processability of Hydrogel-Based Inks for Bioprinted and Moulded Scaffolds
Source: Gels. 2025 Mar 14;11(3):200. doi: 10.3390/gels11030200 (PMC11941948; doi:10.3390/gels11030200)

Effects of PHBV Microparticle Loading on Rheology, Microstructure, and Processability of Hydrogel-Based Inks for Bioprinted and Moulded Scaffolds

Mercedes Pérez-Recalde[1*], Evelina Pacheco[2], Beatriz Aráoz[1*], Élida B. Hermida[1]

[1]Instituto de Tecnologías Emergentes y Ciencias Aplicadas (ITECA), UNSAM, CONICET, Escuela de Ciencia y Tecnología, 1650 San Martín, Buenos Aires, Argentina.

[2] Escuela de Ciencia y Tecnología, UNSAM, 1650 San Martín, Buenos Aires, Argentina

*Corresponding Authors: M. Pérez-Recalde (mrecalde@unsam.edu.ar) and B. Aráoz (baraoz@unsam.edu.ar)

Figure S1: Examples of optical images used to determine diameter particle size.

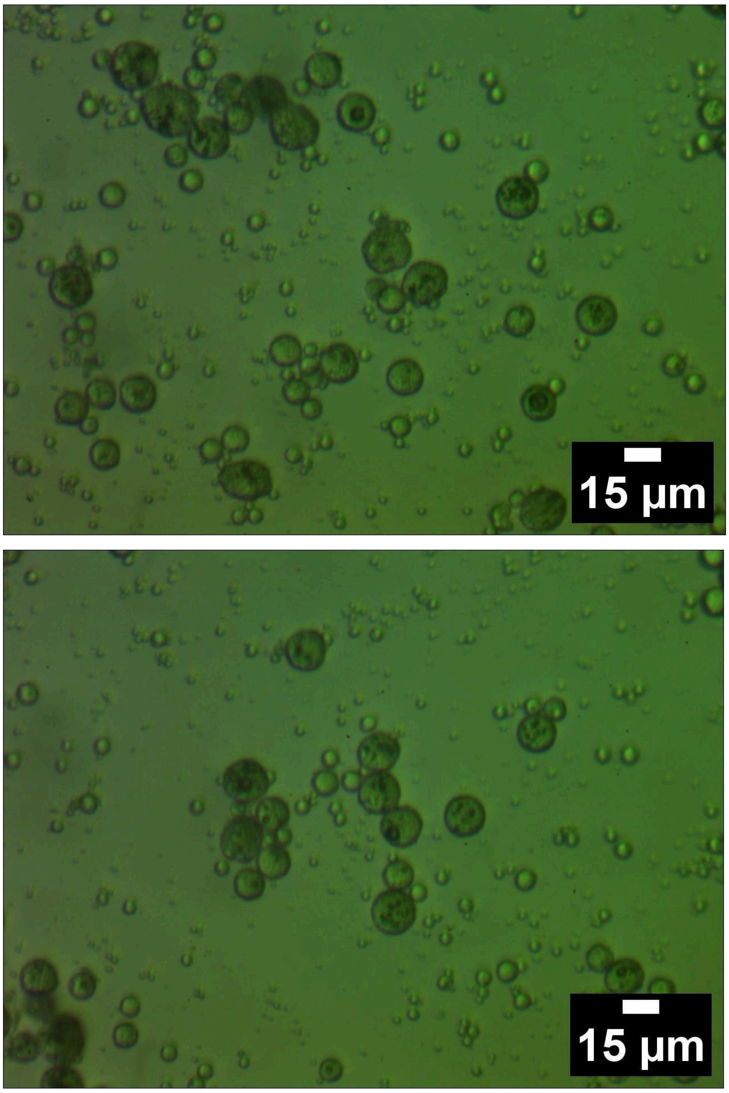

Figure S2: Rheological comparison between Alg-Gel 9%-4.5% and Alg-Gel 9%-4.5% prescrosslinked with calcium 15 mM

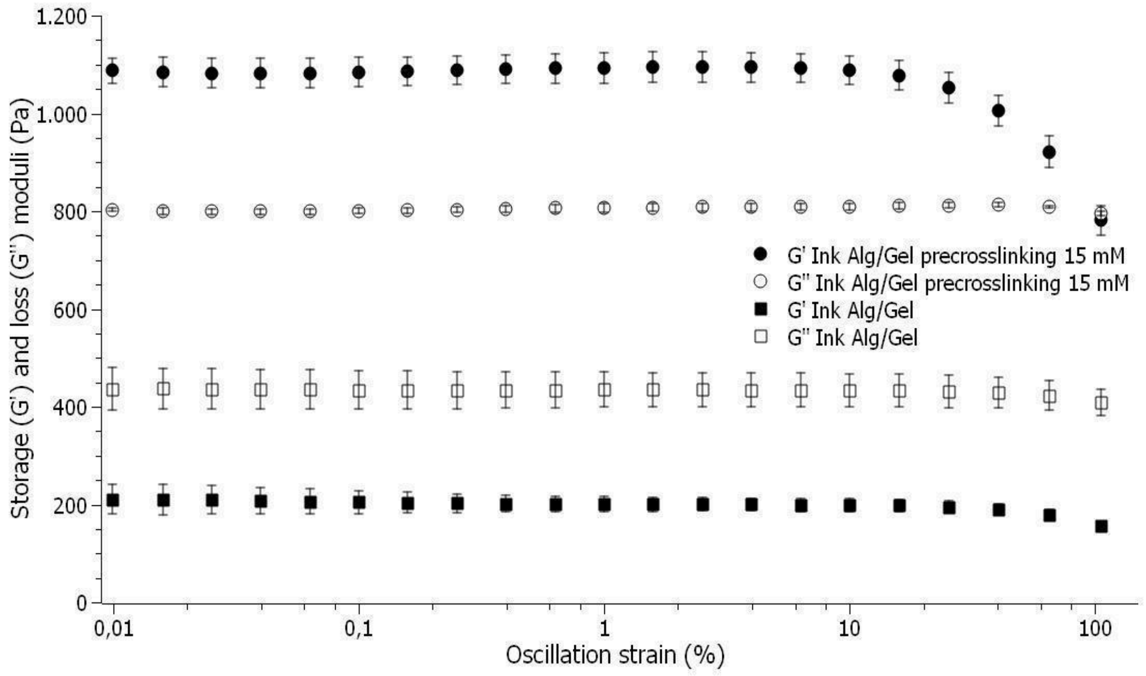

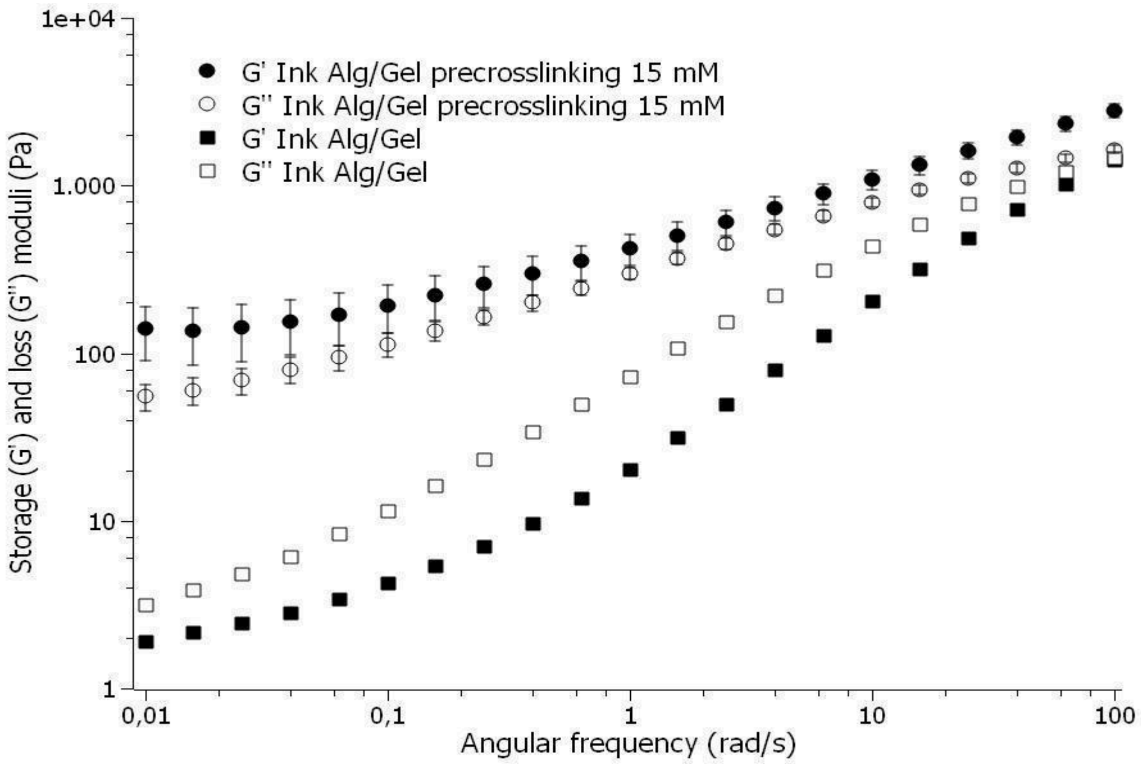

Figure S3: Evaluation of Pr Ink Alg/Gel with different level of calcium precrosslinking (2.5-40 mM)

2,5 mM $CaCl_2$	10 mM $CaCl_2$	15 mM $CaCl_2$	20 mM $CaCl_2$	40 mM $CaCl_2$

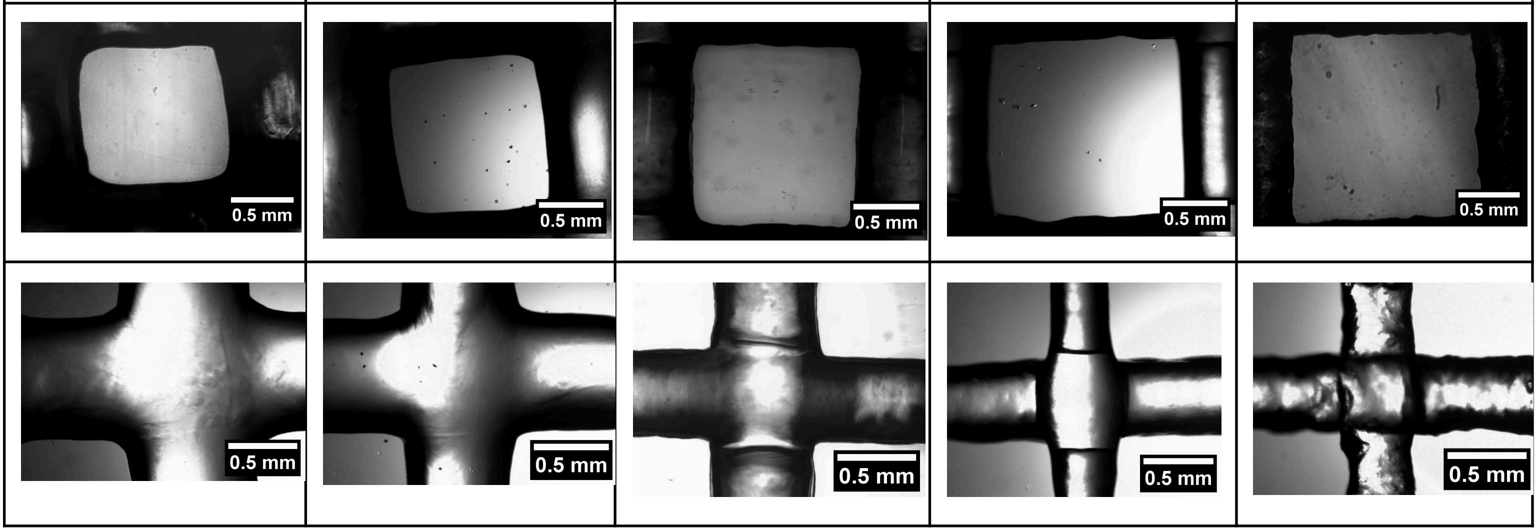

Figure S4: Frequency sweeps for Ink-1.5 and Ink-5.

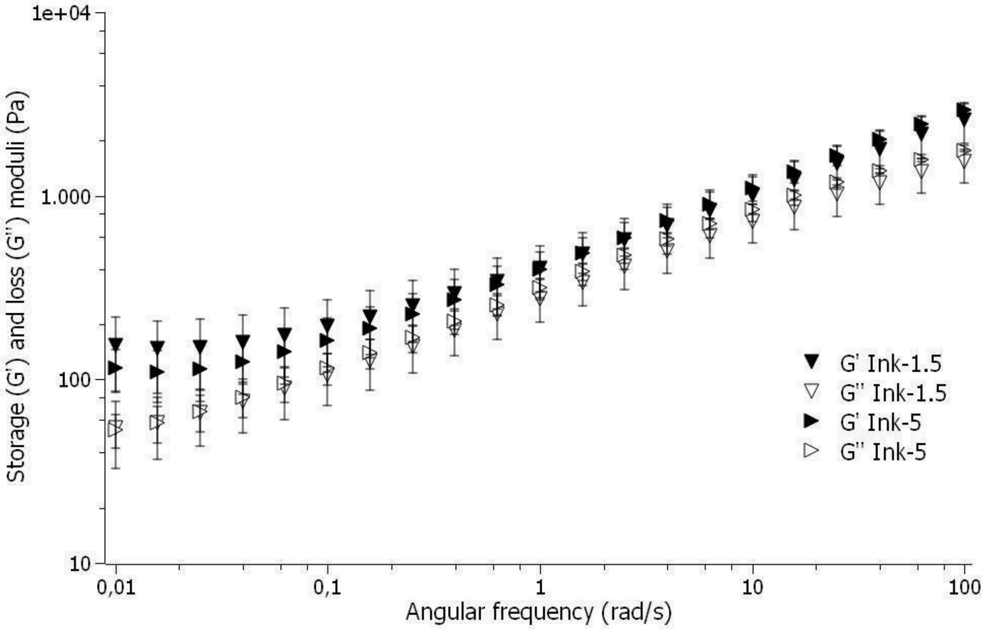

Figure S5: SEM image of cross section of liofilized molded scaffold (produced with Ink-3) after cryogenic fracture.

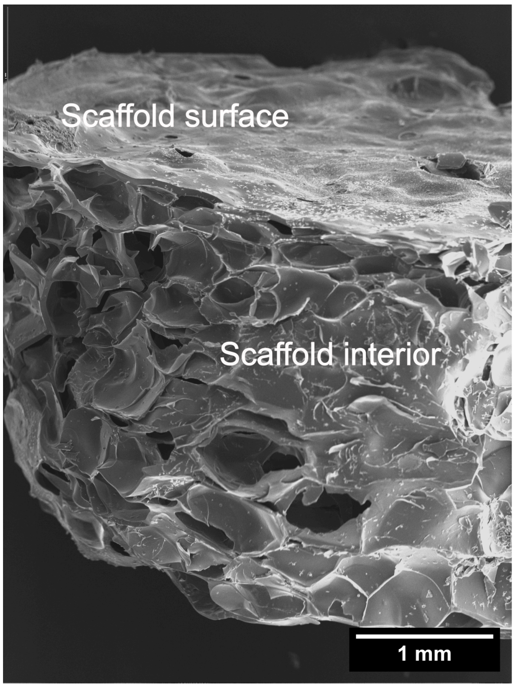

Figure S6: Representative stress vs strain curve of scaffolds produced by moulding of inks containing different concentrations of MP: 0 mg MP/mL (ink), 0.5 mg MP/mL (ink-0.5), 1.5 mg MP/mL (ink-1.5), 3 mg MP/mL (ink-3), 5 mg MP/mL (ink-5), 10 mg MP/mL (ink-10), 15 mg MP/mL (ink-15), and 20 mg MP/mL (ink-20).

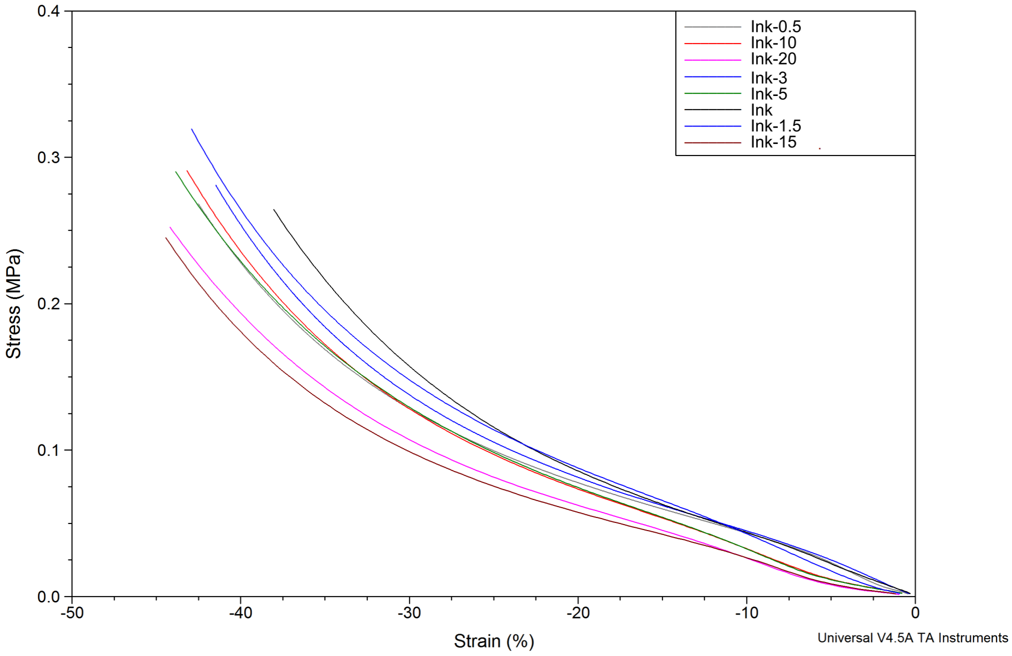

Figure S7: Representative stress vs strain curve of scaffolds produced by bioprinting inks containing different MP concentrations: 0 mg MP/mL (ink) and 3 mg MP/mL (ink-3).

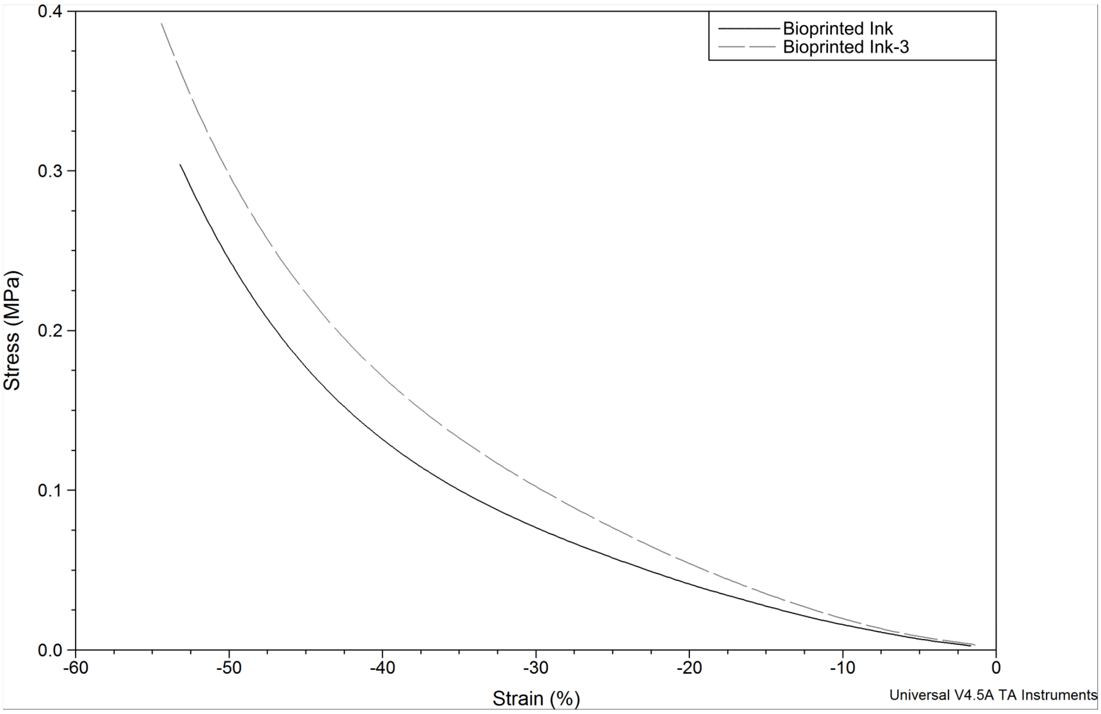

Figure S8: Stability in SBF at 37 °C of moulded (right) and bioprinted (left) scaffolds using PBS or water as a solvent and evaluated as the relative mass change for 7 days

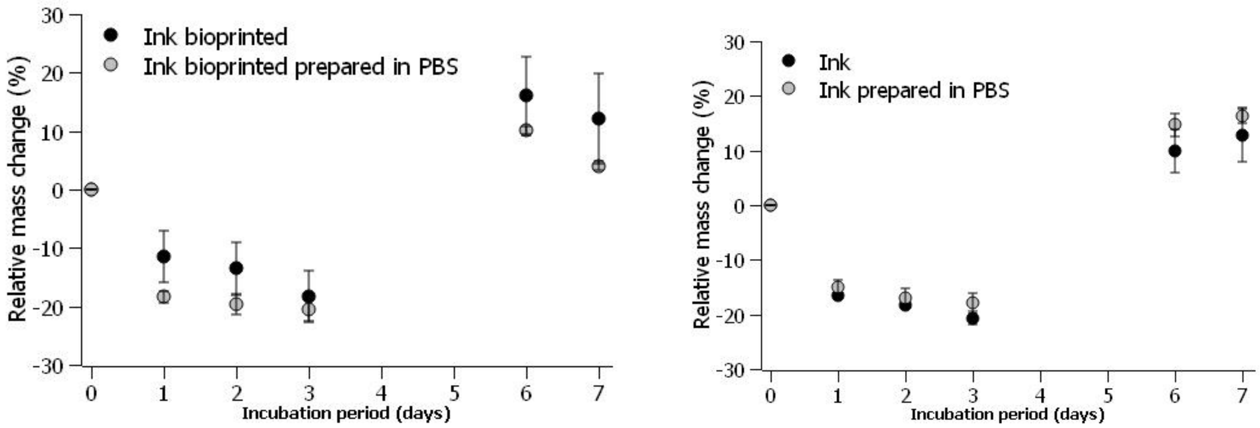

Supplement: Supplementary file 1 [file gels-11-00200-s001.zip › gels-3320815-supplementary.pdf]
